# Perforin and Granzyme B Have Separate and Distinct Roles during Atherosclerotic Plaque Development in Apolipoprotein E Knockout Mice

**DOI:** 10.1371/journal.pone.0078939

**Published:** 2013-10-24

**Authors:** Paul R. Hiebert, Wendy A. Boivin, Hongyan Zhao, Bruce M. McManus, David J. Granville

**Affiliations:** 1 UBC James Hogg Research Centre at the Institute for Heart + Lung Health, St. Paul’s Hospital, Vancouver, British Columbia, Canada; 2 Department of Pathology and Laboratory Medicine, University of British Columbia, Vancouver, British Columbia, Canada; King’s College London School of Medicine, United Kingdom

## Abstract

The granzyme B/perforincytotoxic pathway is a well established mechanism of initiating target cell apoptosis. Previous studies have suggested a role for the granzyme B/perforin cytotoxic pathway in vulnerable atherosclerotic plaque formation. In the present study, granzyme B deficiency resulted in reduced atherosclerotic plaque development in the descending aortas of apolipoprotein E knockout mice fed a high fat diet for 30 weeks while perforindeficiency resulted in greater reduction in plaque development with significantly less plaque area than granzyme Bdeficient mice. In contrast to the descending aorta, no significant change in plaque size was observed in aortic roots from either granzyme Bdeficient or perforindeficient apolipoprotein E knockout mice. However, atherosclerotic plaques in the aortic roots did exhibit significantly more collagen in granzyme B, but not perforin deficient mice. Together these results suggest significant, yet separate roles for granzyme B and perforin in the pathogenesis of atherosclerosis that go beyond the traditional apoptotic pathway with additional implications in plaque development, stability and remodelling of extracellular matrix.

## Introduction

The granzyme B (GzmB)/perforin (Prf1) apoptotic pathway and its role in cytotoxic lymphocyte-mediated apoptosis has been extensively studied since its discovery in the mid-1980’s[1-3]. GzmB is a member of the granzyme serine protease family and has, until recently, been thought to function primarily in CD8+ cytotoxic T cell or natural killer cell mediated apoptosis through a Prf1-dependent mechanism.In this process, upon target cell recognition, GzmB and Prf1 are released towards the target cell whereby GzmB enters the cytoplasm through a mechanism that requires Prf1. GzmB then initiates apoptosis by cleaving multiple different substrates inside of the target cell[1].However, an alternative extracellular, Prf1-independent role for GzmB has been proposed in recent years[1].This is in part due to recent studies showing that GzmB can also be expressed in many other types of immune(macrophages, mast cells, CD4+ T cells, T-regulatory cells, basophils, neutrophils and dendritic cells)and non-immune cells (keratinocytes, pneumocytes and chondrocytes)[4-15]. Therefore, if GzmB-secreting cells do not form immunological synapses with target cells and/or Prf1 is not expressed (eg. mast cells[15], basophils[10]), GzmB may be unable to enter into the cytoplasm and instead accumulate extracellularly. It is this previously underappreciated extracellular activity that is attracting increasing scientific and therapeutic interest. Beginning with observations thatextracellular GzmB is present in elevated amounts in bodily fluidsof patients with chronic inflammatory diseases (eg. plasma[16], bronchoalveolar lavage[17,18], synovial fluid[19], cerebrospinal fluid [20,21]) its extracellular activity is now thought to play an important rolein addition to apoptosis insuch pathologies [1].GzmB can degrade several extracellular matrix (ECM) substrates includingvitronectin, laminin, fibronectin,aggrecan, fibrillin-1, decorin,biglycan and betaglycan[22-28]. GzmB-mediated cleavage of ECMmay contribute to pathology via many mechanisms such asanoikis, altered cell migration, altered endothelial function, enhanced TGF-β bioavailability and loss of tissue architecture and structural integrity [5,23,24,26,29].

 Atherosclerosis has been described as a chronic inflammatory disease, whereby excessive inflammation, apoptosis and ECM degradation contributes to the progression and destabilization of atherosclerotic plaques[30,31]. GzmB-mediated apoptosis has therefore been suggested to play a pathological role in atherosclerosis[32,33]. Additionally, plaque destabilization is characterized by extensive plaque ECM degradation and remodelling. Therefore, as many of these ECM proteinsare well-described GzmB substrates, it is possible that the role of GzmB in atherosclerosis pathogenesis is not limited exclusively to Prf1-dependant intracellular apoptosis, but could potentially include ECM remodelling events as well. Indeed this is supported by numerous clinical studies in which elevated levels of extracellular GzmB have been correlated with disease severity[16,34,35].

 In this study, we investigated the role of both GzmB and Prf1 in the pathogenesis of atherosclerosis. To do so, we made use of the apolipoprotein E (ApoE) knockout (KO) mouse. These mice are known to spontaneously develop atherosclerosis,even when fed a regular chow diet. The development and severity of atherosclerosis in these mice is increased further when they are fed a high fat diet, making the high fat diet-fed ApoE KO mouse a useful model to study advanced atherosclerotic plaque development. We also generated GzmB/ApoE double knockout (DKO) mice and Prf1/ApoE DKO mice to investigate the potential roles of these twoproteins in atherosclerosis. 

## Results

### GzmB and perforin is present in atherosclerotic plaques in ApoE KO mice

**Figure 1:**
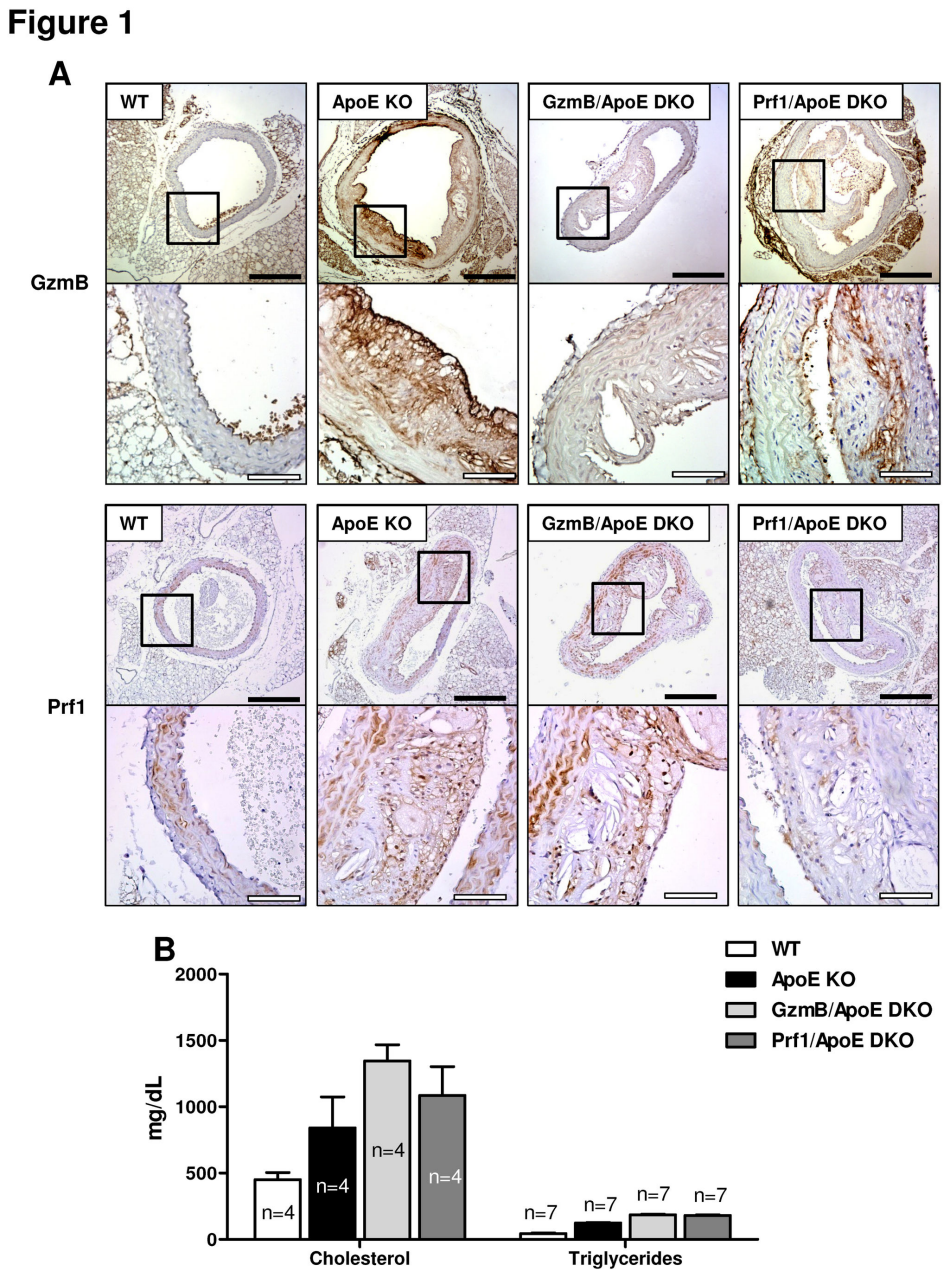
Granzyme B and perforin are present in atherosclerotic plaques from apolipoprotein E knockout mice. (A)Representative images of aorta cross sections from high fat diet-fed wild type (WT),apolipoprotein E knockout(ApoE KO), Granzyme B (GzmB)/ApoE double knockout (DKO) and perforin (Prf1)/ApoE DKOmice stained for GzmB and Prf1. Black scale bars = 400 µm, white scale bars = 100 µm.(B)Neither GzmB nor Prf1 deficiency resulted in a significant difference in circulating levels of cholesterol (n = 4) and triglycerides (n = 7) in ApoE KO mice when fed a high fat diet for 30 weeks.

Increased GzmB levels are observed in human atherosclerotic lesions [33,34]. To determine if GzmB and Prf1 are present in plaques from ApoE KO mice, we performed immunohistochemistry on plaques found in the aortas of wild type (WT) and ApoE KO mice that had been fed a high fat diet for 30 weeks (Figure 1A). As expected, aortas from WT mice fed a high fat diet for 30 weeks exhibited no evidence of atherosclerotic plaque development and minimal GzmB/Prf1 staining (Figure 1A). Conversely, ApoE KO mice possessed numerous atherosclerotic lesions within the aorta that stained strongly for GzmB and also for Prf1 (Figure 1A). 

### Neither GzmB nor Prf1 deficiency affects lipid levels in ApoE KO mice

To further investigate the role of GzmB and Prf1 in the development of atherosclerosis in ApoE KO mice, we generated GzmB/ApoE DKO and Prf1/ApoE DKO mice. Total cholesterol and triglycerides were quantified to determine if GzmB or Prf1 deficiency affected levels of circulating lipids in ApoE KO mice.Plasma isolated from GzmB/ApoE DKOand Prf1/ApoE DKO mice exhibited similar levels of cholesterol and triglycerides to that of ApoE KO mice (Figure 1B), suggesting GzmB and Prf1 do not influence lipid levels in ApoEKO mice when fed a high fat diet for 30 weeks.

### GzmB and Prf1 do not influence plaque size in the aortic roots of ApoE KO mice

**Figure 2:**
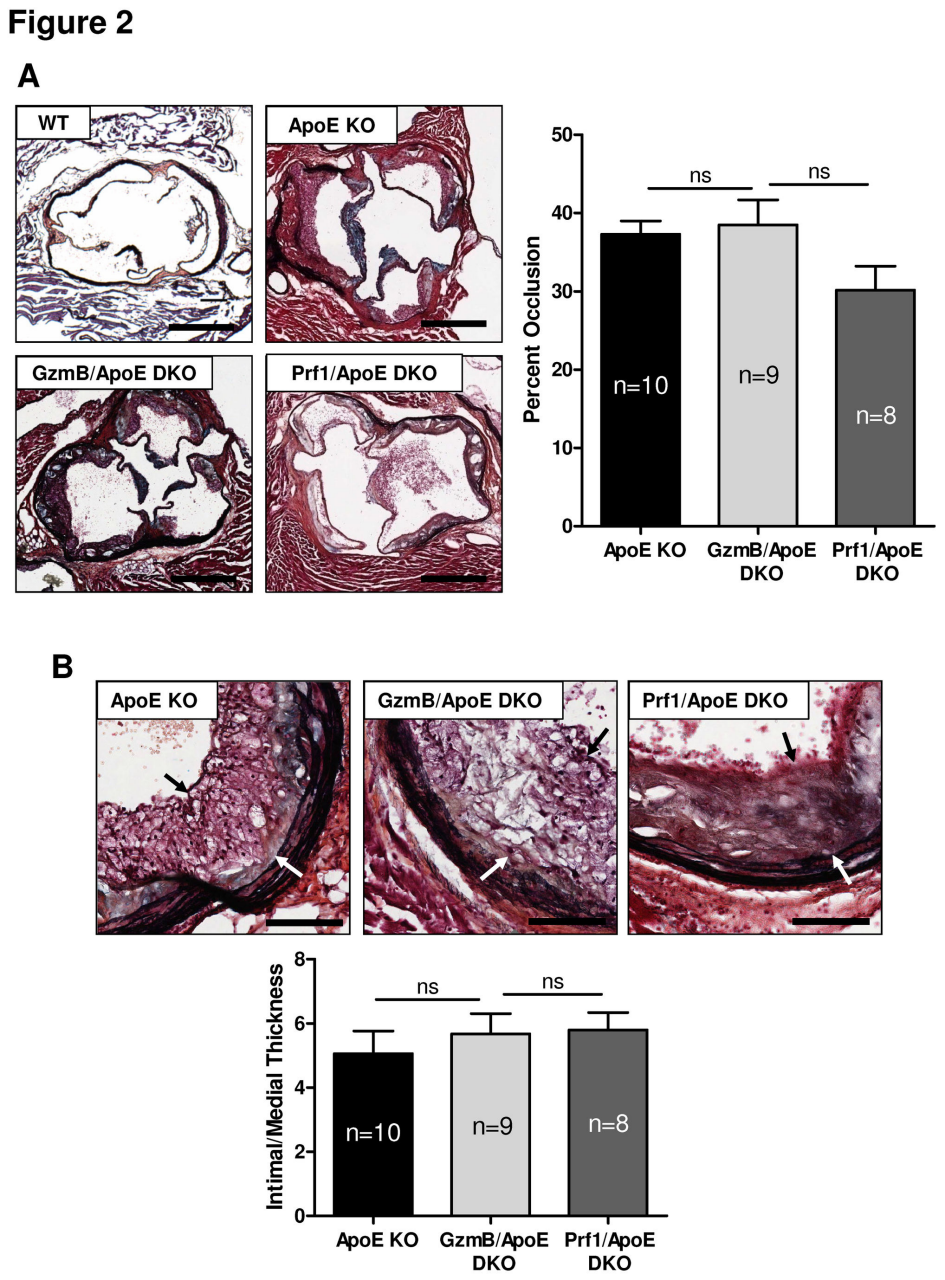
Plaque area in aortic roots from granzyme B or perforin deficient apolipoprotein E knockout mice. (A) Representative images of aortic root cross sections from high fat diet-fed wild type (WT), apolipoprotein E knockout (ApoE KO), granzyme B (GzmB)/ApoE double knockout (DKO) and perforin (Prf1)/ApoE DKO mice stained with Movat’spentachrome. Scale bars = 500 µm. No significant difference in the size of plaque was observed in GzmB/ApoE DKO (n = 9) or Prf1/ApoE DKO mice (n = 8) compared to ApoE KO mice (n = 10). (B) Example images of plaques from aortic roots stained with Movat’spentachrome. The same number of animals were used for these measurements as in panel A. Arrows indicate boundaries of the intimal plaque. Scale bars = 100 µm. No significant difference was detected in the ratio of intimal/medial thickness. ns = not significant (One-way ANOVA with bonferronipost test). Error bars represent SEM.

To determine if GzmB and/or Prf1 influence plaque size, we first investigated the aortic roots of WT, ApoE KO, GzmB/ApoE DKO and Prf1/ApoE DKO mice. As expected, aortic roots from WT mice showed no evidence of plaque development while ApoE KO mice showed clear atherosclerotic lesions when fed a high fat diet for 30 weeks (Figure 2A). Both GzmB/ApoE DKO mice and Prf1/ApoE DKO mice showed no significant difference in the amount of plaquein the aortic roots when compared to ApoE KO mice as measured ether by the percent neointimal plaque area (Figure 2A) or by the ratio of intimal to medial thickness (Figure 2B). 

### Reduced plaque area in the descending aorta in GzmB and Prf1 deficient ApoE KO mice

**Figure 3:**
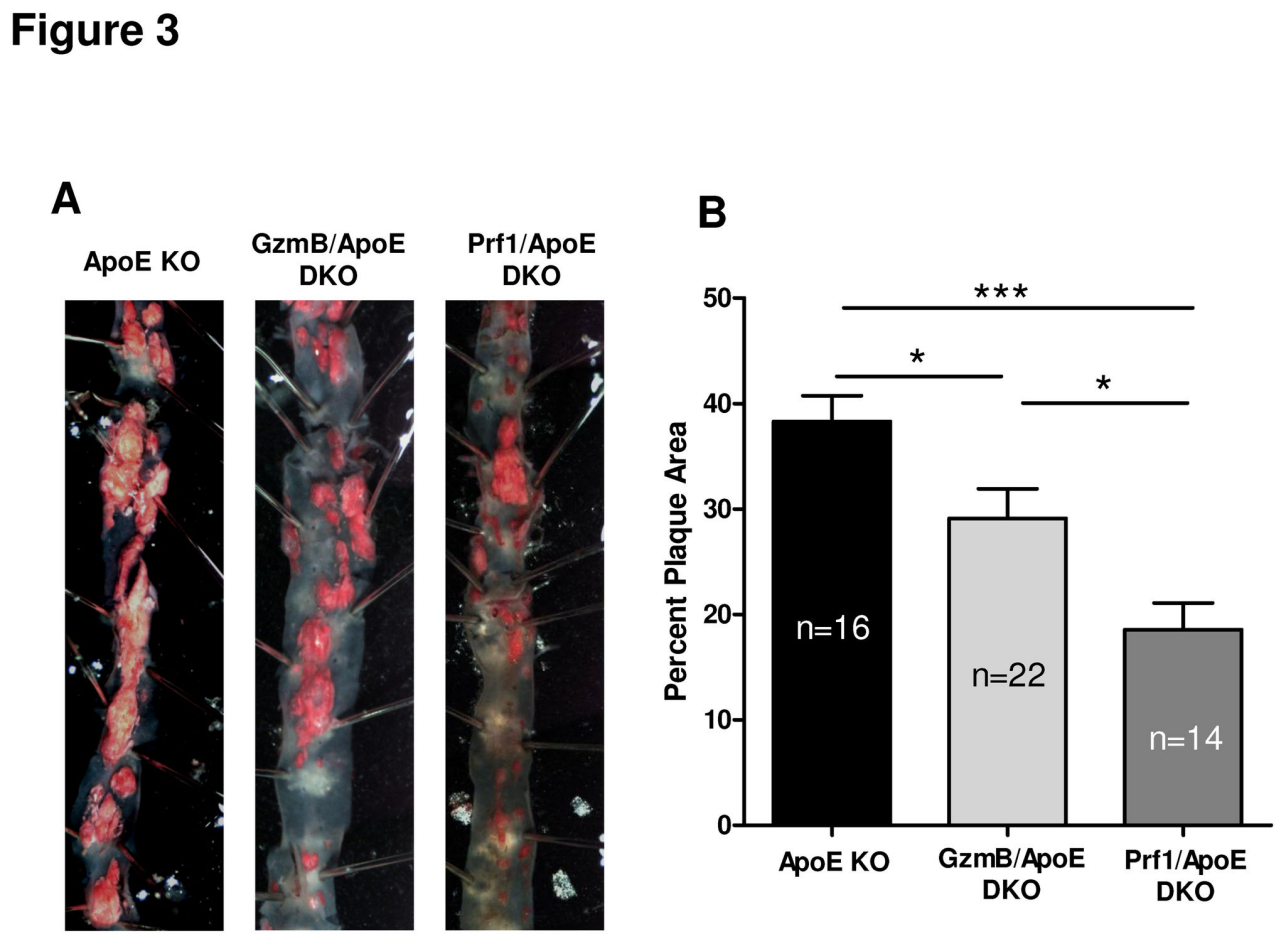
Granzyme B and perforin contribute to plaque development in the descending aorta of apolipoprotein Eknockout mice. (A) Representative images on the descending aorta from high fat diet-fed apolipoprotein E knockout (ApoE KO), granzyme B (GzmB)/ApoE double knockout (DKO) and perforin (Prf1)/ApoE DKO mice stained en face with sudan IV. (B) When plaque area was quantified, GzmB/ApoE DKO mice (n = 22) had significantly reduced plaque area compared to ApoE KO mice (n = 16). Prf1/ApoE DKO mice (n = 14) had significantly less plaque than both the ApoE KO mice and the GzmB/ApoE DKO mice. *P<0.05, ***P<0.005 (One-way ANOVA with bonferonnipost test). Error bars represent SEM.

To further investigate the effect of GzmB and Prf1 on overall atherosclerotic plaque development in the entire aorta, we analysed plaques found in the descending aortas en face stained with sudan IV (Figure 3A). In contrast to our observations in the aortic root sections, we observed a significant decrease in total plaque area in GzmB/ApoE DKO mice compared to ApoE KO mice (Figure 3B). Interestingly, the reduction of plaque area observed in Prf1 deficient mice was also significantly less than that of GzmB deficient mice (Figure 3B). 

### Increased collagen in plaques from GzmB, but not Prf1, deficient ApoE KO mice

**Figure 4:**
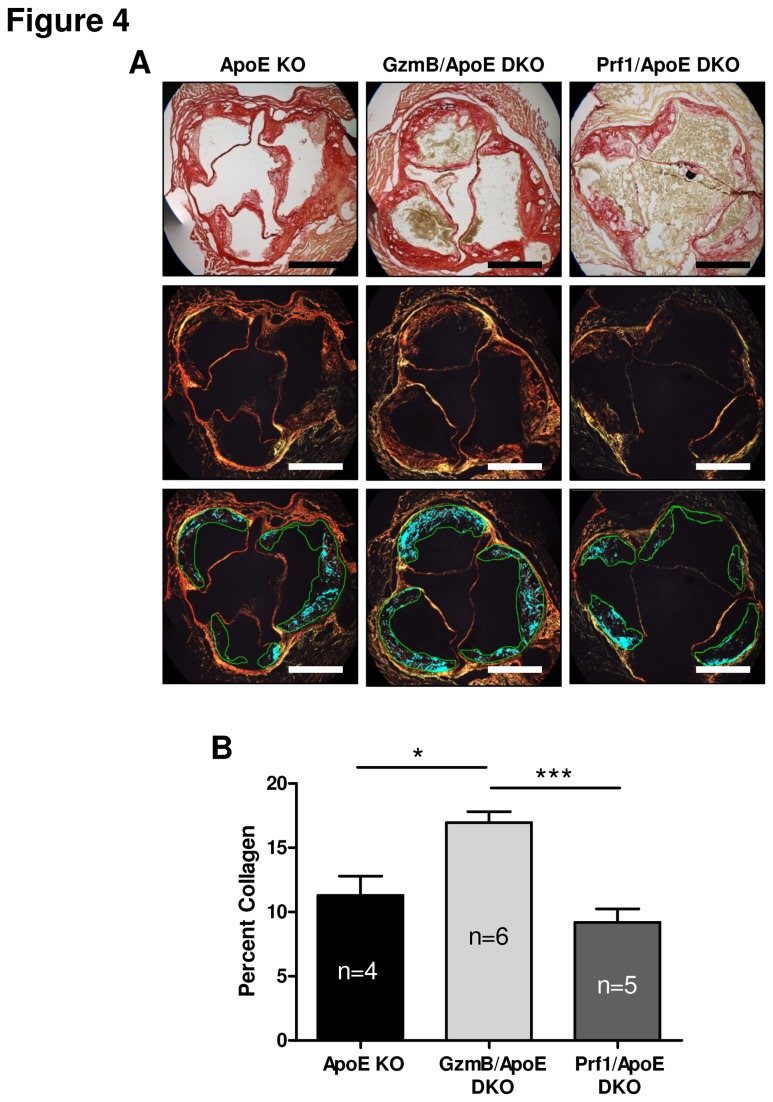
Granzyme B, but not perforin deficiency results in increased collagen content in atherosclerotic plaques. (A) Representative images of aortic root sections stained for collagen using picrosirius red and visualized under bright field or polarized light. Bright field images were used to define the area of plaque and collagen was quantified using the images taken under polarized light. Scale bars = 500 µm. (B) Compared to the high fat diet-fed apolipoprotein E knockout (ApoE KO) mice (n = 4), granzyme B (GzmB)/ApoE double knockout (DKO) mice (n = 6) exhibited increased collagen content in atherosclerotic plaques of the aortic root. Perforin (Prf1)/ApoE DKO mice (n = 5) on the other hand, showed no difference in collagen content compared to ApoE KO mice and significantly less collagen compared to GzmB/ApoE DKO mice. *P<0.05, ***P<0.005 (One-way ANOVA with bonferronipost test). Error bars represent SEM.

Collagen plays a critical role in maintaining atherosclerotic plaque stability (reviewed in 30). To investigate collagen in the plaques of ApoE KO mice, and the affect of GzmB and Prf1 on collagen content, aortic root sections were stained with picrosirius red. Stained sections were visualized under bright field and polarized light (Figure 4A). Compared to ApoE KO mice, GzmB/ApoE DKO mice had significantly greater collagen content in atherosclerotic lesions (Figure 4B). Prf1/ApoE DKO mice, on the other hand,exhibited no significant difference in collagen content compared to ApoE KO mice and had significantly less collagen than GzmB/ApoE DKO mice. Taken together, these results suggest GzmB, but not Prf1, contributes to reduced collagen in atherosclerotic plaques in ApoE KO mice when fed a high fat diet for 30 weeks.

### Altered decorin content in plaques from GzmB and Prf1 deficient ApoE KO mice

**Figure 5:**
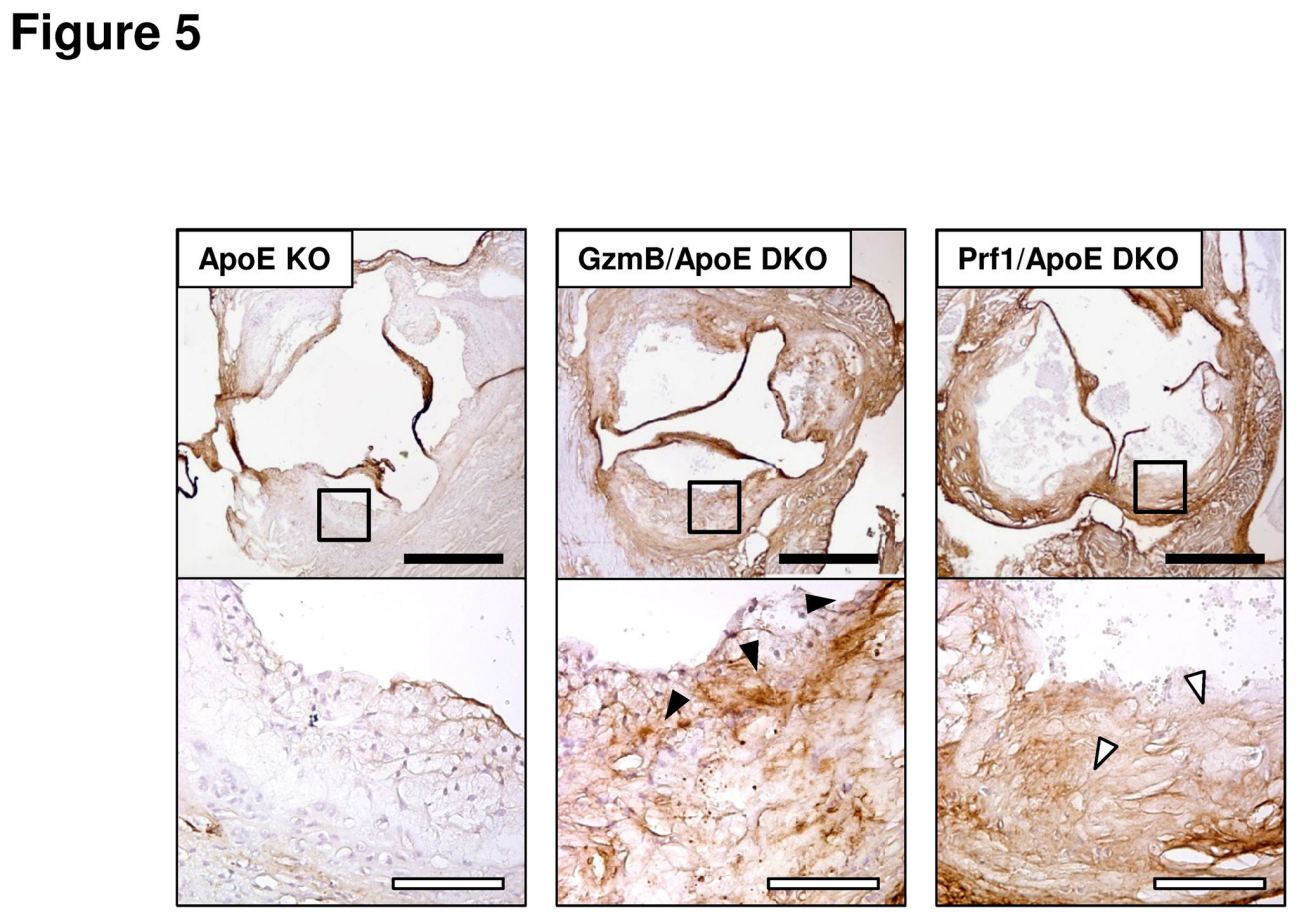
Increased decorin in plaques from granzyme B and perforin deficient apolipoprotein Eknockout mice. Representative images of aortic root sections fromapolipoprotein E knockout (ApoE KO), granzyme B (GzmB)/ApoE double knockout (DKO) and perforin (Prf1)/ApoE DKO mice stained for decorin. Decorin in the GzmB deficient animals was observed near the surface of the plaque in concentrated pockets (black arrowheads) while decorin in Prf1 deficient animals stained more diffusely throughout the plaque (white arrowheads). White scale bars = 50 µm, black scale bars = 500 µm.

 One possible explanation for the difference in collagen content seen in the GzmB/ApoE DKO mice but not the Prf1/ApoE DKO mice is that GzmB is contributing to remodelling of the ECM through a Prf1-independant extracellular mechanism. It has been shown in other models that GzmB contributes to collagen remodelling through the degradation of the proteoglycan, decorin [25,28]. Decorin staining was noticeably weak in the plaques seen in ApoE KO mice (Figure 5). By comparison, plaques from GzmB/ApoE DKO mice exhibited greater decorinimmunopositivity, including areas near the cap region of the plaque (Figure 5, black arrowheads). Interestingly, Prf1/ApoE DKO mice also had increased decorin content in their plaques compared to the ApoE KO mice (Figure 5). Upon closer examination, decorin content appeared tobe differentially distributed in the plaques of Prf1/ApoE DKO mice when compared to GzmB/ApoE DKO mice with reduced decorin in the fibrous capand a more diffuse staining pattern (Figure 5, white arrowheads). 

### GzmA, T cells and macrophages in plaques from GzmB and Prf1 deficient ApoE KO mice

**Figure 6:**
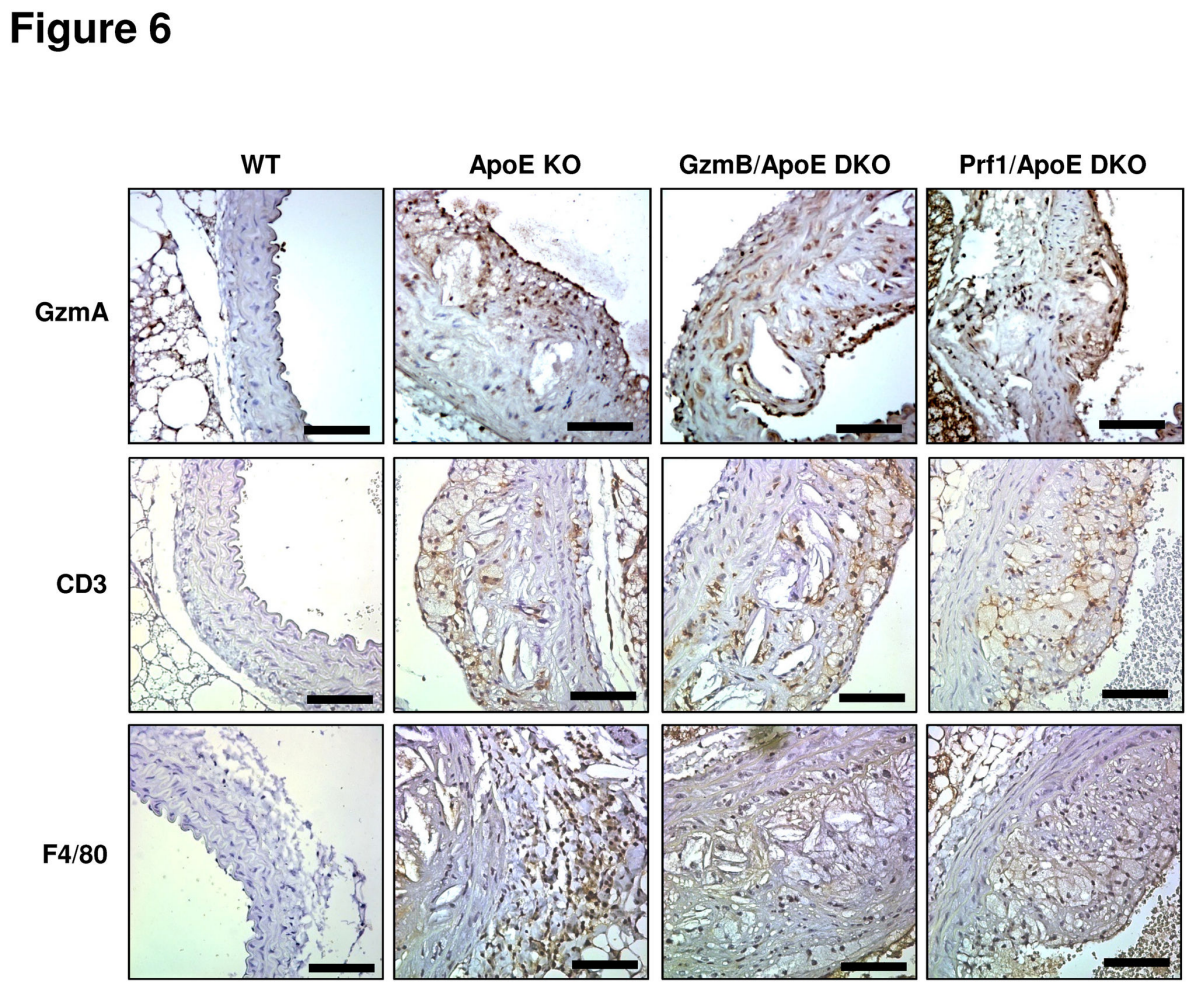
Expression of granzyme A, T cells and macrophages in atherosclerotic plaques. Representative images of wild type (WT), apolipoprotein E knockout (ApoE KO), granzyme B (GzmB)/ApoE double knockout (DKO) and perforin(Prf1)/ApoE DKO mouse aortas stained for (A) granzyme A, (B) CD3 and (C) F4/80. Scale bars = 100 µm.

Granzyme A (GzmA), similar to GzmB can be expressed during chronic inflammation and is capable of Prf1-dependant and independent activity that can potentially influence ECM remodelling [36-38] as well as cytokine processing and release [39-42]. We investigated the presence and localization of GzmAin the plaques of mice to see if ApoE, GzmB or Prf1 deficiency influenced the levels of GzmA in the aortic wall. As shown in Figure 6, WT mouse aortas exhibited minimal positive staining for GzmA in the aortic wall compared to the plaques in ApoE KO mice which demonstrated noticeablestaining for GzmA (Figure 6).Staining was observed throughout the plaque in all groups of mice and no obvious differences were observed in the composition and localization of GzmA.Plaques from GzmB/ApoE DKO and Prf1/ApoE DKO mice also stained positive for GzmA. These results suggest that GzmB and/or Prf1do not affect the expression of GzmA in plaques from ApoE KO mice and that the phonotypic differences observed in these mice is not due to altered expression of GzmA. Similarly, CD3 positive T cells and F4/80 positive macrophages were also detected in the plaques from all groups of mice (Figure 6). Plaques from ApoE KO mice exhibited positive staining for F4/80 in the area in and around the adventitia while all groups contained macrophage foam cells in the intimal plaque that stained lightly for F4/80. CD3 immunopositivitywas similar in all groups of mice containing plaques with no obvious differences in staining patterns observed. 

## Discussion

The role of GzmB in cardiovascular diseases has been a topic of increasing attention in recent years [32,43,44].Prf1-dependant, GzmB-mediated apoptosis has been the primary mechanism investigated in this regard and recent studies have suggested evidence of this pathway as an important mechanism in vulnerable plaque formation[32-34].While absent in the normal vessel and vessels with mild atherosclerosis, GzmB is abundant in vessels with advanced disease and GzmB expression is associated with increased disease severity and plaque instability[34]. Macrophages, cytotoxic cells and smooth muscle cells display GzmB positivity and GzmB co-localizes to TUNEL-positive foam cells undergoing apoptosis [33,34]. The intracellular GzmB inhibitor, protease inhibitor 9, is decreased in atherosclerotic lesions, suggesting an increased susceptibility of resident cells to GzmB-induced cell death [33]. High plasma GzmB levels have also been linked to plaque instability and increased cerebrovascular events as soluble GzmB levels are highest in individuals with thin, rupture-prone fibrous caps[16]. In the present study, both GzmB and Prf1were found to exert pathological roles in the ApoE KO mice. The data suggests that GzmB and Prf1exert differential roles in atherosclerosis, influencing plaque composition and plaque development, respectively. 

The role of Prf1 in GzmB internalization followed by the initiation and execution of apoptosis is well-described[1]. However, more recently it has become clear that granzymes are more than pro-apoptotic proteases and many additional Prf1-independent, extracellular roles for GzmB including the ability to degrade the ECM have been identified [22,23,25-27,29,45]. In this study, consistent with previous studies [28], GzmB was associated with reduced collagen birefringence. This observation appeared to be Prf1-independent as we did not observe this phenomenon in the Prf1 deficient mice. Only GzmB deficiency, not Prf1 deficiency, resulted in increased collagen content in plaques found in the aortic roots of ApoE KO mice, suggesting that Prf1-independent, extracellular GzmB activity contributes to ECM remodelling in atherosclerotic plaques. These results are similar to that observed in a mouse model of abdominal aortic aneurysm, where GzmB deficiency, but not Prf1 deficiency was protective against mortality due to aneurysm rupture [23]. Similarly, the use of an extracellular GzmB inhibitor, serpina3n, also protected against aneurysm rupture due to increased adventitial collagen [28].

While multiple reports have confirmed the inability of GzmB to degrade collagen [22,26,29], other ECM componentssusceptible to GzmB-mediated cleavage caninfluence collagen remodelling[22,23,25-29,45]. The proteoglycan decorininteracts with collagen and has a profound influence on collagen organization, spacing and tissue tensile strength [46]. Previous work also showed that decorin overexpression reduced atherosclerosis development in ApoE KO mice, supporting a protective role for decorin in vascular diseases[47]. Decorin degradation by GzmB has been shown to contribute to a loss of collagen density in the adventitia of the aorta during abdominal aortic aneurysm, contributing to aneurysm rupture, exsanguination and mortality in mice[28]. GzmB also degrades decorin in the skin, where it is believed to contribute to age-related skin frailty and a loss of collagen organization[25].In the present study, increased decorin was observed in plaques from GzmB/ApoE DKO mice compared to ApoE KO mice, supporting the hypothesis that GzmB degrades decorin *in vivo*. Interestingly, when compared to ApoE KO mice, Prf1/ApoE DKO mice also showed greater staining for decorin.The absence of Prf1 may have a number of currently unknown consequences that could account for this observation including effects on immune regulation, recruitment of immune cells, proteases and other granzymes into the plaque. It is known that multiple granzymes can influence cytokine release and processing both intracellularly and extracellularly[39-41,48-52]. Although beyond the scope of this study, the absence of Prf1 during chronic inflammatory diseases such as atherosclerosis may have a considerable impact on any processed/expressed pro-inflammatory cytokinesthat are activated intracellularly by granzymes and could conceivably influence the nature of inflammation(including further granzyme expression) in atherosclerotic plaques further affecting ECM remodelling. While staining for CD3 positive T cells and F4/80 positive macrophages failed to demonstrate clear differences between the GzmB and Prf1 deficient mice, other factors including the activation and/or subset of these cells could potentially be affected by Prf1 deficiency although this remains speculation. It is unknown if differences in immune mediators accounts for the decorin observations made in the Prf1/ApoE DKO mice however future studies on the effects of Prf1 deficiency on inflammation during chronic inflammatory disease is warranted. As other proteases may also degrade decorin, it is possible that Prf1 deficiency also affects the expression of other immune-secreted proteases which could impact decorin levels. Altered decorin expression in plaques from Prf1 deficient mice is another possible explanation. Nevertheless, differences in staining patterns were observed between the GzmB and Prf1 deficient animals, suggesting a specific GzmB-mediated effect on decorin in the plaques of ApoE KO mice during atherosclerosis development.Further work is required to better understand the pro-inflammatory and proteolytic mechanisms involved in decorin/collagen remodelling in the plaques of ApoE KO mice and the ultimate effects of these events on atherosclerosis disease progression.

The current study also provides evidence thatPrf1contributes to the incidence and development of atherosclerosis in the descending aorta through mechanisms that are independent of GzmB. While GzmB deficiency resulted in significantly reduced plaque area in the descending aorta of ApoE KO mice, Prf1-deficient micehad an even greater reduction that showed significantly less plaque area than the GzmB deficient animals. One possible explanation for this is the action of other granzymes. Cytotoxic granules contain not only GzmB and Prf1 but a number of other granzymes as well. In humans there are 5 different granzymes while 11 exist in mice. GzmB deficiency would therefore not affect Prf1-mediated entry of other granzymes into the cytoplasm of target cells where they could continue to perform their alternative functions. These functions may include and are not necessarily limited to apoptosis, cytokine processing and cytokine release. While there is still much to be discovered concerning the possible intracellular roles of these other granzymes, any intracellular contribution to disease development would presumably remain in GzmB deficient mice, but abolished when Prf1 is absent. In the present study, we observed similar staining for GzmA in the plaques of ApoE KO, GzmB/ApoE DKO and Prf1/ApoE DKO mice.The exact role of GzmA and its influence on inflammation and plaque development in atherosclerosis remains to be investigated. However, given the well-documented role for IL-1β in inflammation and atherosclerosis combined with the known role for GzmA in the production of this cytokine and others, it is plausible that GzmA could be contributing to atherogenesisas well [39-42].

Other studies have also investigated the role of GzmB and Prf1 in atherosclerosis.One study by Viswanathan*et al* showed that GzmB/ApoE DKO mice exhibit a trend towards reduced plaque development compared to ApoE KO mice in a model of aortic allograft vasculopathy[53]. On the other hand, a different study using the low density lipoprotein receptor (LDLr) KO model showed that Prf1/LDLr DKO mice were not protected against atherosclerosis pathogenesis compared to LDLrKO controls [54]. This appears contradictory to our study but the discrepancies may be indicative of the different models used, differencesin age and time spent on a high fat diet (reviewed in 55). For example,diet, sex, genotype and agehave been shown to influence atherosclerotic plaque size in mice [56]. Our studies were carried out with mice being fed a high fat diet for 30 weeks compared to the LDLrKO study where mice were fed a high fat diet for only 16 weeks.Age is known to be an important risk factor for atherosclerosis and the influence of both GzmB and Prf1 may be greater when examining a more severe plaque such as those seen in aged mice[57]. This is also consistent to that observed in the clinic where the highest levels of GzmB are associated with plaque instability and immediately after plaque rupture[16,34,35]. As such, GzmB-mediated ECM degradation may play a more profound role in advanced atherosclerosis and plaque rupture compared to the early stages of the disease. This is certainly true for aneurismal rupture whereby extracellular GzmB inhibition is protective while Prf1-deficiency had no effect on rupture or survival[23,28]. Improved models of plaque rupture would also aid greatly in furtherexamining this hypothesis.

In conclusion, both Prf1 and GzmB contribute to the pathogenesis of atherosclerosis in ApoE KO mice. The present study suggests that Prf1 and GzmB exert unique roles in the onset and progression of atherosclerosis. 

## Materials and Methods

### Ethics statement

All research studies involving the use of animals were conducted with the approval of the University of British Columbia (UBC) Animal Care Committeeand in compliance with The Canadian Council on Animal Care.Animals were monitored continuously for signs of illness or distress which would occasionally arise in the form of severe skin lesions [25]. Any animal deemed to exhibit signs of suffering were euthanized for humane reasons as per UBC guidelines.

### Mice

GzmBKO, ApoEKO and Prf1 KO mice were purchased from The Jackson Laboratory (Bar Harbor, ME). GzmB/ApoEDKO and Prf1/ApoEDKO mice where then bred in house at the Genetically Engineered Models facility at the UBC James Hogg Research Centre (Vancouver, BC).At 6-8 weeks of age WT, ApoEKO, GzmB/ApoEDKO and Prf1/ApoEDKO mice began a high fat diet (21.2% fat, 0.2% cholesterol, Harlan Teklad, Madison, WI) for 30 weeks. At the time of harvest, mice were weighed and euthanized by isofluorane/CO_2_ inhalation. Blood was then collected by cardiac puncture. Vessels were perfused with saline at a constant pressure of 100mmHg using a pressurized tubing system for 5min or until no blood was observed at the incisionin the right atria. Hearts and aortas were then collected and fixed in 10% formalin. 

### Total Cholesterol and Triglyceride Quantification

To determine total cholesterol levels in mouse plasma, the Cholesterol E Enzymatic ColormetricAssay (Wako, Richmond VA) was utilized. In brief, a 1:100 dilution of mouse plasma and appropriate cholesterol standards were added to a colour reagent containing cholesterol ester hydrolase, cholesterol oxidase, peroxidise, 4-aminoantipyrine 3,5-Dimethoxy-N-ethyl-N-(2-hydroxy-3-sulfopropyl)-aniline sodium salt and ascorbate oxidase. Cholesterol esters were converted to free cholesterol and fatty acids and cholesterol was oxidized to generate hydrogen peroxide which then reacts to form a blue pigment. After a 5min incubation at 37°C, the blue pigment was measured at an absorbance of 600nm.Four mice per group were used for these experiments.

To determine total triglycerides in mouse plasma the Triglyceride Colormetric Assay Kit was utilized (Cayman Chemical Company, Ann Arbor MI). A triglyceride enzyme mixture was added to a sodium phosphate assay buffer which then reacts to form a purple pigment to be read at 540nm. Plasma was added to the plate at a 1:16 ratio.Seven mice per group were used for these experiments.

### Aortic root and en face quantification of plaque area

Fixed aortic root sections cut 5 µm thick were stained with Movat’spentachrome and images were taken at 10Xmagnification. The area of the plaque was quantified and expressed as the percent occlusion of the vessel lumen (vessel lumen area was calculated by tracing the base of the plaque above the media around the entire vessel). Lesion area was traced and quantified using the imaging software ImageProPlus® version 4.5.0.29 for Windows (Media Cybernetics Inc, Rockville MD). The intimal / medial thickness measurements were performed as described previously [58] by measuring the mean intimal thickness and normalizing to the mean medial thickness. These measurements were performed on ApoE KO (n = 10), GzmB/ApoE DKO (n = 9) and Prf1/ApoE DKO mice (n = 8). For en face analysis, aortas were trimmed for the removal of excess fat and tissue surrounding the aorta. Vessels were then pinned to a paraffin tray and tissue was covered in staining solution (0.5% sudan IV (Fisher Scientific, Waltham, MA), 35% ethanol and 50% acetone) for 15min. Solution was removed and replaced with 80% ethanolfor 5 min for decolourization. Aortas were then washed in dH_2_O for 1h, photographed and plaque area traced using ImageProPlus®.These measurements were done on ApoE KO (n = 16), GzmB/ApoE DKO (n = 22) and Prf1/ApoE DKO mice (n = 14).

### Collagen Quantification

To examine collagen content, 5 µm aortic root sections were stained in picrosirius red solution and images were taken at 10X magnification under bright field and polarized light. The area of interest was identified using bright field images and then applied to the images taken under polarized light, where collagen was then quantified by colour segmentation.The imaging software ImageProPlus® was used for these analyses.These measurements were done on ApoE KO (n = 4), GzmB/ApoE DKO (n = 6) and Prf1/ApoE DKO mice (n = 5).

### Immunohistochemistry

Paraffin sections (5 µm) were treated with xylene and rehydrated with ethanol and PBS. The tissues were treated in boiling citrate buffer and endogenous peroxidase was quenched in 3% H_2_O_2_. Ten percent rabbit serum or 10% goat serum was used for blocking after which rabbit anti-GzmA (kind gift from Dr. Julian Pardo, Zaragoza, Spain and Dr. Markus Simon,Freiburg, Germany),goat anti-GzmB (Abcam, Cambridge, MA),rabbit anti-perforin (Cell Signaling, Boston MA), rabbit anti-CD3 (Abcam), rat anti-F4/80 (AbDSerotec, Raleigh, NC), goat anti-decorin (R&D Systems, Minneapolis MN) in 10% serum was incubated overnight at 4°C. The secondary antibody biotinylatedrabbit anti-goat, goat anti-rabbit or goat anti-rat(Vector Laboratories, Burlingame, CA) was incubated at room temperature for 30 minutes in 5% serum. ABC reagent was used as directed (Vectastain Elite ABC kit, PK-6100, Vector Laboratories) and was visualized with DAB (Vector Laboratories). Tissue was then counterstained with hematoxylin.
